# Supplementary material for: Cultivation and Imaging of S. latissima Embryo Monolayered Cell Sheets Inside Microfluidic Devices
Source: Bioengineering (Basel). 2022 Nov 21;9(11):718. doi: 10.3390/bioengineering9110718 (PMC9687954; doi:10.3390/bioengineering9110718)
Supplement: Supplementary file 1 [file bioengineering-09-00718-s001.zip › Supplementary_Information_I1.pdf]

Supplementary Information:
Cultivation and imaging of *S. latissima* embryo monolayered cell sheets inside microfluidic devices

Thomas Clerc, Samuel Boscq, Rafaele Attia, Gabriele S. Kaminski Schierle, Bénédicte Charrier, and Nino F. Läubli

Content:

Supplementary Figures:

Figure S1: Effect of sample density on egg release and growth behaviour

Figure S2: Microscopy setup with controlled environmental conditions

Figure S3: Comparison of daily growth rates and blade shapes between on-chip cultures with and without liquid renewal and open space

Figure S4: Blue and red light spectra for physiological investigation

Figure S4: Time-lapse growth tracking using microspheres

Captions for Supplementary Movies:

Movie M1: Transition from oogonium to egg

Movie M2: Growth of mature embryos

Supplementary Data:

Data D1: Data used for the production of the presented graphs (see separate file)

Supplementary Figures

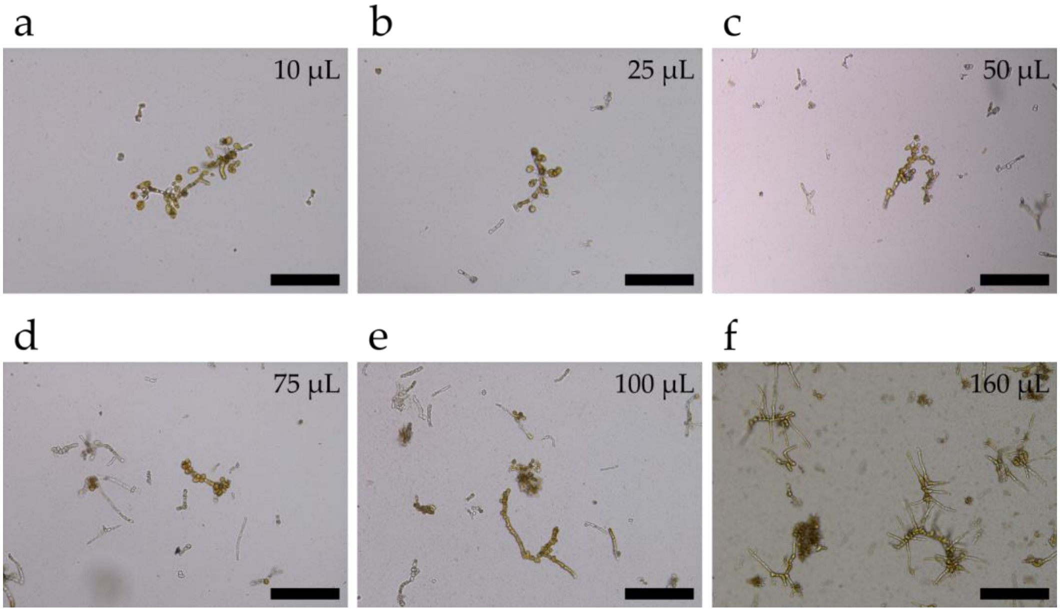

Figure S1. Different concentrations of *Saccharina latissima* gametophytes left to induce gamete release and embryo growth. From the initial crushed gametophyte stock culture, (a) 10, (b) 25, (c) 50, (d) 75, or (e) 100 µL were taken and diluted with 2 mL of natural sea water inside Petri dishes. Egg release was drastically reduced at higher density of gametophytes even though the stage of the gametophyte should still allow oogenesis. (f) If left to grow in unfavourable conditions, the cells (that normally differentiate into an egg-producing oogonium) form more gametophyte cells instead of eggs. Specimens were cultured under white light for (a) – (e) 2 weeks or (f) 3.5 weeks. Scale bars: 250 µm.

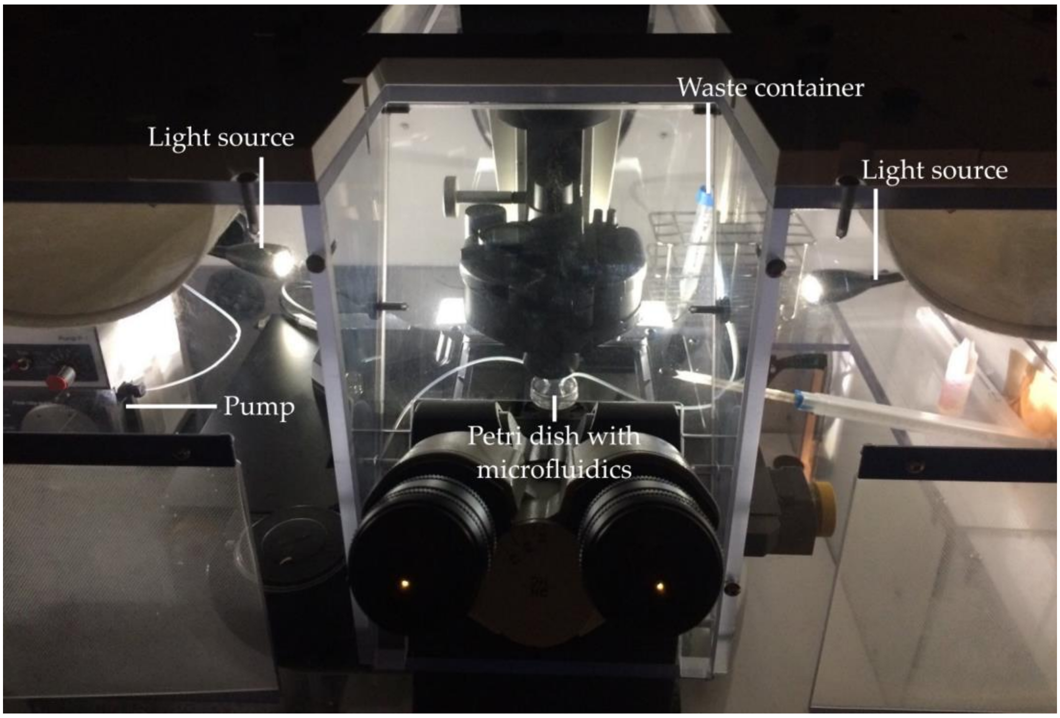

Figure S2. Microscopy setup with controlled environmental conditions. The setup consists of an inverted microscope incubation chamber. Light intensity equal to standard culturing conditions is provided *via* two lateral LEDs. The glass-bottom Petri dish containing either the samples directly or the microfluidic device with the specimens is highlighted at the centre of the image. For experiments requiring automatic liquid renewal, the microfluidic device was connected to a pump (left side) while the liquid waste was collected on the right side through tubing connected to the device's outlet.

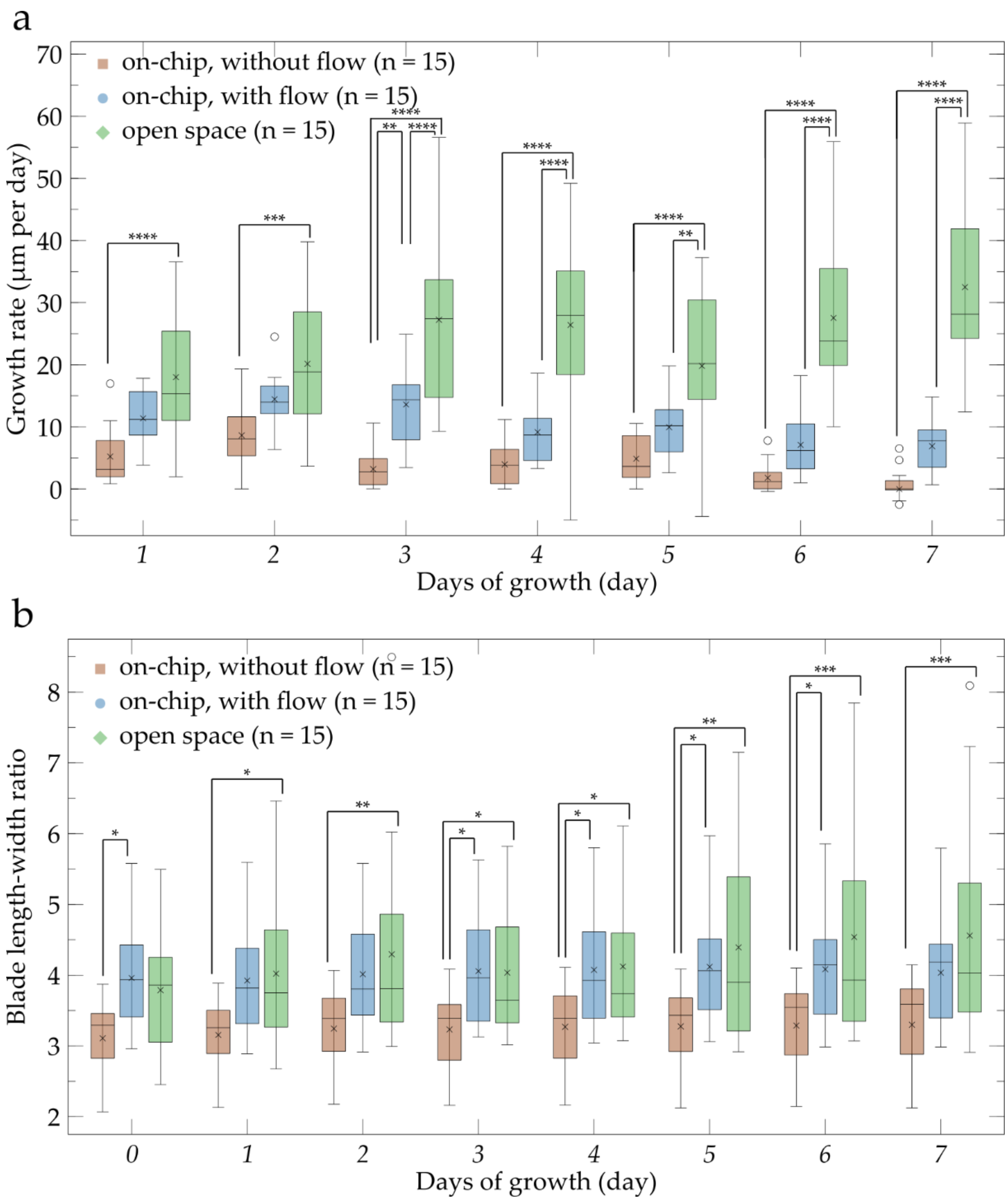

Figure S3. Comparison of daily growth rates and blade shapes between on-chip cultures with and without liquid renewal and open space. (a) Growth rates of specimens cultured in open space, *i.e.*, in glass bottom Petri dishes, are on most days significantly higher than for specimens growing inside microfluidic devices with and without liquid renewal. (b) Specimens growing inside the device with liquid renewal show similar blade shapes with regard to their length-width ratio as samples growing outside the device, while specimens inside the chips without liquid renewal are less elongated. Asterisks indicate statistically significant differences in growth rate and blade length-width ratio between the culturing conditions on specific days as analysed using with two-way ANOVAs (see Materials and Methods).

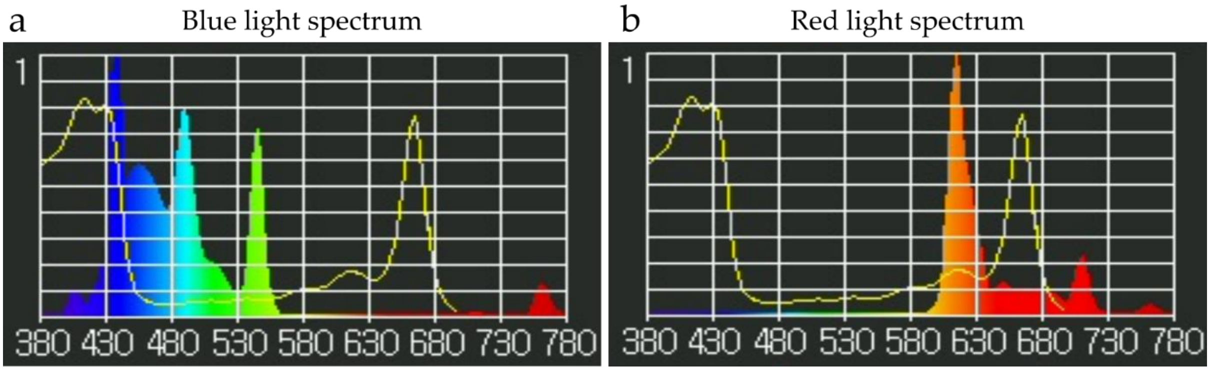

Figure S4. Blue and red light spectra for physiological investigation.

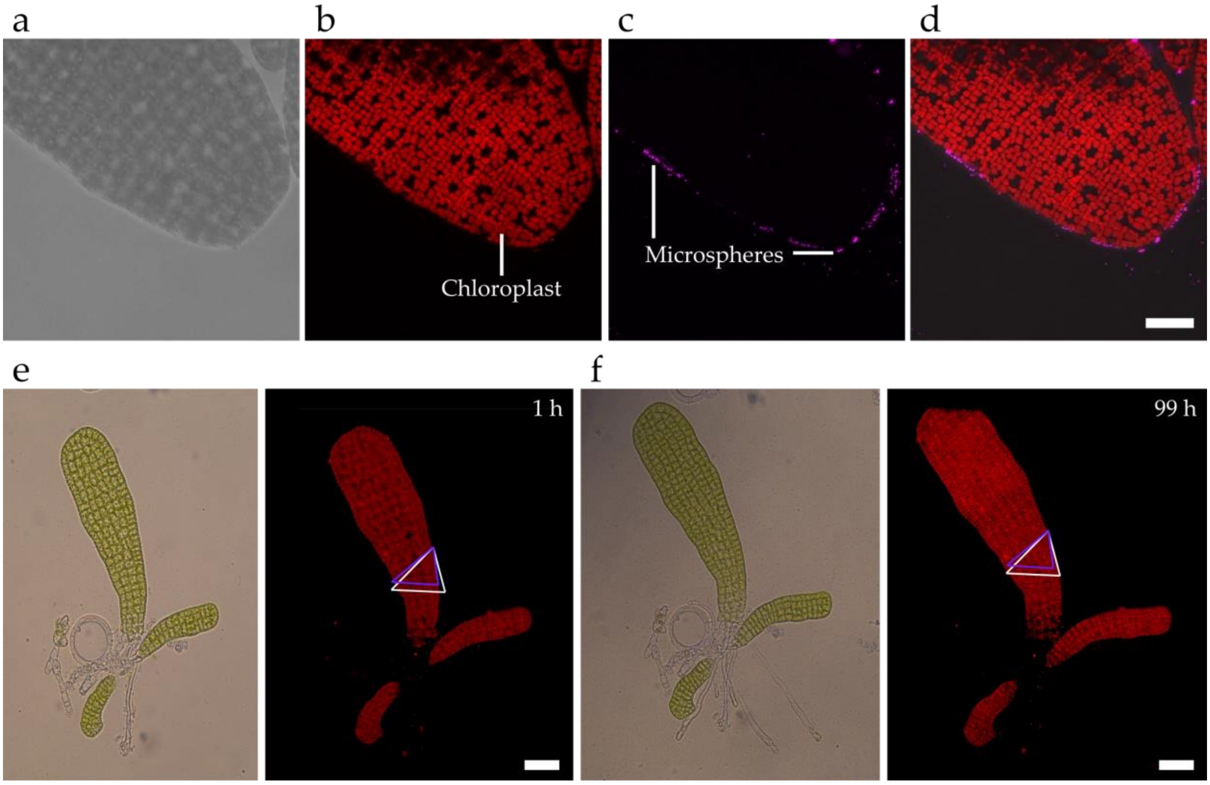

Figure S5. Confocal microscopy image of a monolayered lamina labelled with fluorescent beads for live tracking. (a) Brightfield image of the monolayered lamina. (b) Autofluorescence of the chloroplasts. (c) 200 nm red fluorescent microspheres attached to the lamina. Only the beads stuck at the outline of the lamina were imaged, thereby making sure that they are in the same focal plane. (d) Merged microscopy image of the fluorescence channels showing the chloroplasts (red) and the functionalised microspheres (purple). (e, f) Brightfield and fluorescence images of a monolayered lamina labelled with fluorescent microspheres growing over ~ 4 days. The triangle joins 3 red beads at 1 h (blue) and 99 h (white). The larger white triangle illustrates the higher distance between the beads and, therefore, growth of the embryo in this area. Scale bars: (a-d) = 25 µm; (e, f) = 50 µm.

Supplementary Movie Captions:

Movie characteristics: Movie duration: 20 sec, with 5 image acquisitions per sec (total 100 images). Each image acquisition corresponds to one hour of time-lapse microscopy (one image every hour). Total duration in real-time is approximately 4 days.

Movie S1. Time-lapse movie showing a growing monolayered sheet of a Saccharina embryo and a maturating gametophyte. Note the hatching of 3 eggs over time, each in less than one hour.

Movie S2. Time-lapse movie showing a growing monolayered sheet of a Saccharina embryo differentiating rhizoids. Note one of the rhizoids pushing the small embryo lamina in the bottom left side of the movie, thereby showing that all the algal material grows in a single plane.
